# Ovarian sensitivity index-based nomogram for predicting clinical pregnancy outcomes in patients with diminished ovarian reserve undergoing *in vitro* fertilization or intracytoplasmic sperm injection

**DOI:** 10.3389/fmed.2025.1618552

**Published:** 2025-06-27

**Authors:** Feng-Xia Liu, Ka-Li Huang, Shan-Jia Yi, Hui Huang, Ming-Hua Shi, Xue-Fei Liang

**Affiliations:** Reproductive Hospital of Guangxi Zhuang Autonomous Region, Nanning, China

**Keywords:** diminished ovarian reserve, *in vitro* fertilization, intracytoplasmic sperm injection, ovarian sensitivity index, nomogram

## Abstract

**Background:**

Predicting clinical pregnancy outcomes in patients with diminished ovarian reserve (DOR) undergoing *in vitro* fertilization/intracytoplasmic sperm injection (IVF/ICSI) remains challenging owing to the unique characteristics of this patient group. Therefore, this study aimed to leverage existing predictive models for pregnancy outcomes while integrating innovative strategies to develop and validate a visualization-based predictive model specifically designed for patients with DOR undergoing IVF/ICSI treatment.

**Methods:**

This retrospective study analyzed data from 448 patients with DOR who underwent IVF/ICSI at Guangxi Zhuang Autonomous Region Reproductive Hospital from January 2019 to August 2023. We developed and internally validated a nomogram incorporating the ovarian sensitivity index (OSI), age, and controlled ovarian hyperstimulation (COH) protocol to predict clinical pregnancy outcomes. Receiver operating characteristic (ROC) analysis, univariate and least absolute shrinkage and selection operator (LASSO) regression analyses, and multivariate logistic regression were used to construct the model. The optimal cut-off value of the OSI for predicting clinical pregnancy was 1.135.

**Results:**

Through multivariate analysis, age, OSI, and COH protocol were identified as independent predictors. The developed nomogram demonstrated good discrimination with an area under the ROC curve of 0.744, along with satisfactory calibration and clinical utility.

**Conclusion:**

The developed nomogram can accurately predict clinical pregnancy outcomes in patients with DOR undergoing IVF/ICSI, potentially assisting clinicians in personalized counselling and improving outcomes in this challenging patient population.

## 1 Introduction

Diminished ovarian reserve (DOR) is characterized by a reduction in the quantity and quality of oocytes. Clinically, DOR is characterized by decreased levels of the anti-Müllerian hormone (AMH) and antral follicle count (AFC), along with elevated levels of baseline follicle-stimulating hormone (bFSH). Currently, the diagnostic criteria for DOR lack full standardization (1). According to the 2022 clinical consensus on the diagnosis and treatment of diminished ovarian function in China, DOR is diagnosed when a patient exhibits <5–7 antral follicles in both ovaries, serum AMH levels of <1.1 ng/mL, and bFSH levels of ≥10 IU/L across two consecutive menstrual cycles (2). DOR affects approximately 20% of the infertile population, with an increasing prevalence and earlier onset (3). As the quantity and reproductive potential of oocytes diminish, natural conception becomes more challenging for patients with DOR. These individuals usually rely on assisted reproductive technologies (ART), such as *in vitro* fertilization/intracytoplasmic sperm injection (IVF/ICSI)-embryo transfer, to conceive. However, patients with DOR undergoing IVF/ICSI encounter several obstacles, including fewer retrieved oocytes, fewer high-quality embryos, and higher rates of cycle cancellation, all of which significantly contribute to reduced pregnancy rates compared with those with normal ovarian reserves (4). Enhancing IVF/ICSI outcomes in patients with DOR remains a pivotal challenge in the field of reproductive medicine, and the lack of personalized methods for assessing pregnancy outcomes in these patients undergoing IVF/ICSI further complicates clinical management. Therefore, developing an accurate and personalized clinical prediction model to evaluate pregnancy outcomes in patients with DOR undergoing IVF/ICSI is essential.

Studies have identified multiple predictive factors associated with pregnancy outcomes following IVF/ICSI treatment, including age; body mass index (BMI); duration, type, and cause of infertility; baseline hormone levels; AMH; AFC; fertilization method; number of oocytes retrieved; and embryo quality (5, 6). Considering these factors, several studies have developed predictive models for pregnancy outcomes in patients with infertility undergoing IVF/ICSI (7, 8). However, owing to the unique characteristics of patients with DOR, these models have some limitations within this group. For this specific population, age, ovarian responsiveness, ovarian stimulation protocols, number of oocytes retrieved, and embryo quality are considered major factors influencing assisted pregnancy outcomes (9–12). The ovarian sensitivity index (OSI), which assesses ovarian responsiveness by calculating the ratio of the total number of mature oocytes to the total amount of exogenous gonadotropins used during the treatment cycle, provides a novel perspective for predicting pregnancy outcomes in patients with DOR undergoing IVF/ICSI (13). Compared with other ovarian responsiveness indicators, OSI, as a ratio-based index, effectively correlates a patient’s response to gonadotropins with the number of oocytes retrieved and accommodates the diversity of age, AMH levels, and ovarian stimulation protocols. It has demonstrated high consistency in its predictive efficacy for pregnancy outcomes, making it important for predicting pregnancy outcomes in patients with DOR undergoing IVF/ICSI (14). A pressing need exists to establish personalized prediction models specifically tailored to the DOR, incorporating more precise factors. Such models could enable reproductive endocrinologists to better assess treatment prospects, develop reasonable treatment plans, and improve the clinical pregnancy outcomes of patients with DOR while reducing their physical and mental burdens.

According to our preliminary literature review, no previous studies have incorporated OSI into predictive models for evaluating pregnancy outcomes in patients with DOR undergoing IVF/ICSI treatment. Therefore, this study aimed to leverage existing predictive models for pregnancy outcomes while integrating innovative strategies, including the adoption of OSI, to develop and validate a visualization-based predictive model specifically designed for patients with DOR undergoing IVF/ICSI treatment. This model is anticipated to provide novel references for clinical diagnosis and treatment decisions, enhance assisted reproductive management for patients with DOR, improve fertility quality, and be of critical importance for advancing clinical practice and academic research in this field.

## 2 Methods

### 2.1 Study population

In this retrospective study, clinical data from patients with DOR who underwent ICSI/IVF at the Guangxi Zhuang Autonomous Region Reproductive Hospital between January 2019 and August 2023 were analyzed. All data were extracted using an ART management system.

### 2.2 Inclusion criteria

The inclusion criteria were AFC of <5–7 and serum AMH level of <1.1 ng/mL (2). The AFC assessment was performed by transvaginal ultrasound examination during the early follicular phase (days 2–4) of the menstrual cycle. The antral follicles measuring 2–9 mm in diameter in both ovaries were counted. All ultrasound examinations were performed by experienced sonographers to minimize inter-observer variation.

### 2.3 Exclusion criteria

In this study, the exclusion criteria included hydrosalpinx, adenomyosis, endometriosis, history of intrauterine adhesions, and uterine malformations.

### 2.4 Controlled ovarian hyperstimulation protocols

#### 2.4.1 Gonadotropin-releasing hormone agonist protocol

A subcutaneous injection of 1.25 mg of gonadotropin (Gn)-releasing hormone agonist (GnRH-a) was administered during the mid-luteal phase, followed by Gn initiation 14 days later. The starting Gn dose was determined based on the patient’s age, AFC, AMH level, BMI, and prior ovarian response. Follicular monitoring was conducted 4–5 days after Gn initiation, assessing follicle size, follicle number, and serum levels of luteinizing hormone (LH), estradiol, and progesterone. Gn doses were adjusted according to follicular growth. Gn administration was discontinued when at least three follicles reached ≥18 mm in diameter. In the evening, 2000 IU of human chorionic gonadotropin (HCG) and 250 μg of recombinant HCG were administered. Oocyte retrieval was performed 34–38 h later, with embryo transfer conducted on day 3 (D3) and blastocyst transfer on days 5–6 (D5/D6).

#### 2.4.2 Gonadotropin-releasing hormone antagonist protocol

Gn was initiated on day 2 or 3 of menstruation, with follicular development monitored according to the long protocol. GnRH antagonist (GnRH-A) at a dose of 0.25 mg was started on day 6 of Gn treatment or when follicles reached 14 mm and was continued until the HCG day. GnRH-A was discontinued when at least three follicles measured ≥17 mm or two reached ≥18 mm. In the evening, 2000 IU of HCG and 250 μg of recombinant HCG were administered. Oocyte retrieval was performed 34–38 h later, followed by embryo transfer on day 3 (D3) or blastocyst transfer on days 5–6 (D5/D6).

#### 2.4.3 Embryo assessment and transfer strategy

D3 embryos were graded using a 4-grade morphological system based on cell number, uniformity, and fragmentation percentage (15). Grades I-II represented optimal quality (uniform cells, <20% fragmentation), while Grades III-IV indicated progressively declining quality with increasing fragmentation, irregularity, or degeneration. Embryos with ≥7 cells and Grade I-II morphology were classified as good quality.

Blastocysts (D5/D6 embryos) were evaluated using the Gardner scoring system, which assigns grades based on three parameters: developmental stage (1–6, from early blastocyst to fully hatched), inner cell mass (ICM) quality (A–C, from tightly packed to sparse), and trophectoderm (TE) quality (A–C, from cohesive to sparse epithelium) (16). Only Stage 3–6 blastocysts with distinguishable cellular components received ICM and TE quality grades. Blastocysts graded as AB, BA, or BB were considered good-quality embryos.

The transfer protocol prioritized single embryo transfer using the highest quality available embryo, with a maximum limit of 2 embryos per cycle. The number of embryos to be transferred was also determined based on embryo quality, patient characteristics, and clinical judgment.

### 2.5 Data collection

#### 2.5.1 Baseline characteristics

Baseline clinical data of patients with DOR, including age, BMI, infertility type, bFSH, AMH, COH protocol, total Gn dose, OSI, number of retrieved oocytes, number of metaphase II (MII) oocytes, number of normally fertilized oocytes, fertilization protocol, endometrial thickness, embryo stage, number of transferred embryos, and embryo grade, were collected.

#### 2.5.2 Outcome measure

In clinical pregnancy, an intrauterine gestational sac with a fetal heartbeat is present at 28 days post-transfer.

### 2.6 Statistical analysis

All data analyses were performed using R software (v4.3.3).

#### 2.6.1 OSI prediction evaluation

The OSI was calculated using the following formula (17):


$$\begin{array}{l} \mathrm{OSI}=(\text{Number of oocytes retrieved}\times 1,000)\\ /\text{Total}\;\mathrm{Gn}\;\text{dosage}\;(\mathrm{IU}). \end{array}$$

The number of oocytes retrieved represents the total count of oocytes obtained during the retrieval procedure, and the total Gn dosage represents the cumulative dose of gonadotropins (FSH and/or LH) administered throughout the stimulation cycle, measured in International Units (IU). The predictive power of OSI for forecasting pregnancy rates was evaluated using ROC analysis with the pROC package (version 1.18.5) in R. This analysis helped in determining the optimal cut-off value, which was subsequently used to stratify patients into high and low OSI groups for further analysis.

#### 2.6.2 Data preprocessing

All variables were converted into unordered categorical variables to simplify the analysis and enhance interpretability. OSI was categorized into high and low based on the threshold determined by ROC analysis. Age was categorized into groups (<35, 35–37, 38–40, 41–42, 43–44, and ≥45 years) based on recommendations from the Chinese Society of Reproductive Medicine (18). These age groups reflected recognized clinical thresholds for the IVF/ICSI procedure. BMI was categorized into groups (< 18.5, 18.5–22.9, and ≥23 kg/m^2^) based on recommendations from the World Health Organization (2004) guidelines (19). Pregnancy outcomes were defined as zero for non-pregnancy and one for clinical pregnancy. Infertility type was categorized as primary infertility, secondary infertility, or others (no history of infertility). COH protocols were classified as GnRH-a or GnRH-A protocol, the fertilization protocols were recorded as IVF and ICSI, and embryo grade was assigned as low- or good-quality embryos.

#### 2.6.3 Baseline clinical characteristics

Baseline clinical characteristics were analyzed using the compareGroups (version 4.9.1) package in R. The compareGroups package automatically assessed the normality of continuous variables using the Shapiro–Wilk test and applied appropriate statistical tests accordingly. Normally distributed continuous variables are expressed as mean ± standard deviation and were compared using t-tests. Non-normally distributed continuous variables are expressed as median [Q1; Q3] and were compared using non-parametric tests (Mann–Whitney U test). Categorical variables are expressed as proportions and were analyzed using the chi-square test or Fisher’s exact test as appropriate, with statistical significance defined as *p* < 0.05.

#### 2.6.4 Model construction and visualization

Initial variable screening was conducted using univariate logistic regression, and variables with *p* < 0.1 were selected for further analysis. Subsequently, predictor selection was refined using LASSO regression analysis with the glmnet package (version 4.1.8). The LASSO model was tuned using 10-fold cross-validation to determine optimal lambda values (which represent the regularization parameter that controls the extent of L1 regularization, influencing the degree to which coefficients shrink toward zero to prevent overfitting and enhance model simplicity). Two lambda values were evaluated: lambda.min (the value that gives the minimum mean cross-validated error) and lambda.1se (the largest value of *λ* such that the error is within one standard error of the minimum). Variables with non-zero coefficients at the selected lambda were retained for subsequent analysis. The final lambda selection was based on the criterion within one standard deviation (lambda.1se) to balance model simplicity and predictive performance. Multivariate logistic regression analysis was performed to identify significant predictors of clinical pregnancy using a forward selection method with a significance threshold of *p* < 0.05. This method was selected for its efficiency in iteratively adding significant predictors to the model. Finally, a predictive model was constructed and visually represented using a nomogram with the rms package (version 6.8.0).

#### 2.6.5 Model validation

The nomogram model was rigorously validated internally using the bootstrap method (including 2,000 bootstrap samples). Additionally, the validation process included (a) assessing the discrimination ability by calculating the area under the ROC curve (AUC) and 95% confidence interval (CI) using the pROC packages (version 1.18.5). An AUC exceeding 0.7 signifies acceptable discriminatory power. (b) The prediction accuracy was determined through calibration curves and the Hosmer–Lemeshow (H–L) test using the rms package (version 6.8.0). The well-aligned calibration curves indicated good agreement between the predicted probabilities and observed outcomes. In the H–L test, P ˃ 0.05 suggests no significant difference between the observed and predicted values, indicating a good model fit. (c) Evaluation of clinical utility using decision curve analysis (DCA) with the rmda package (version 1.6). The DCA curve demonstrates the net benefit of using the model to predict pregnancy outcomes at different risk thresholds compared to not using the model.

## 3 Results

### 3.1 Study process

**Figure 1:**
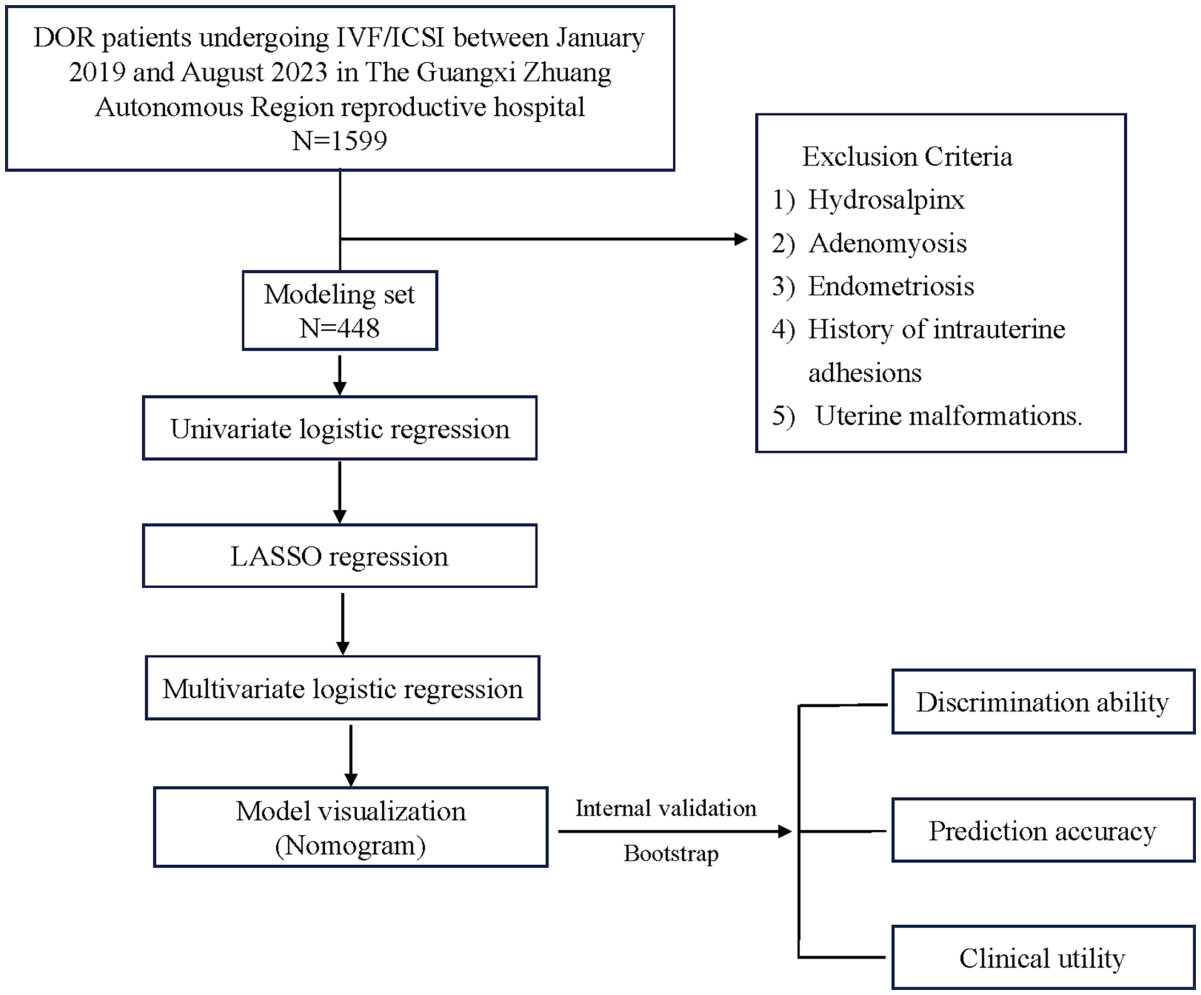
Flow chart of the study.

Clinical data were collected from 1,599 patients with DOR in this study. Following the application of the exclusion criteria, a total of 448 patients were included in the modelling set. This study process is detailed in Figure 1.

### 3.2 OSI prediction evaluation

**Figure 2:**
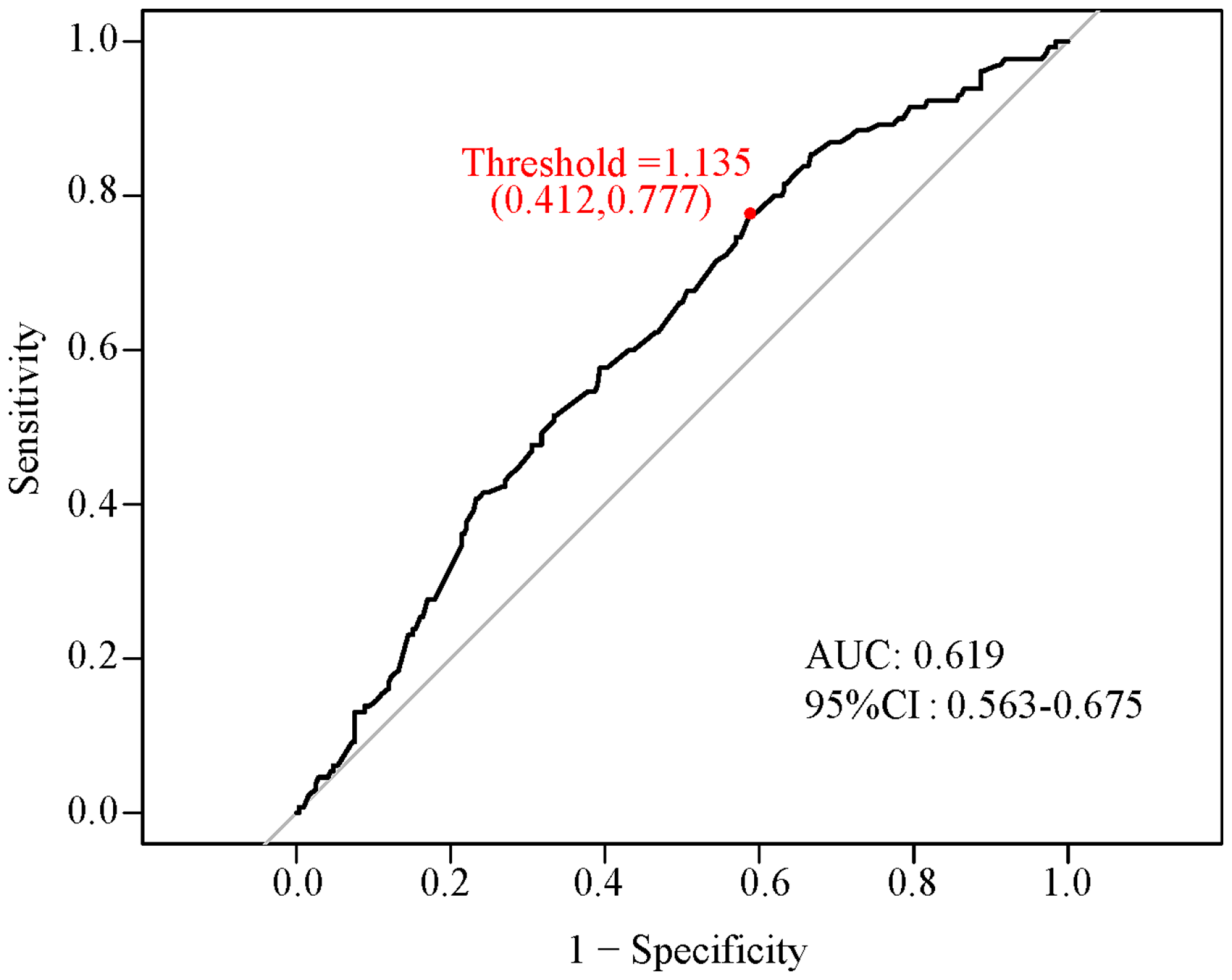
ROC curve showing the optimal threshold of OSI. The X-axis represents 1-specificity, and the Y-axis represents sensitivity. The area under the curve (AUC) was equal to 0.619 (95% CI: 0.563–0.675). The option cut-off threshold equals 1.135 (sensitivity = 0.777, specificity = 0.412). ROC, receiver operating characteristic; OSI, ovarian sensitivity index; CI, confidence interval.

The predictive value of OSI for IVF/ICSI clinical pregnancy outcomes was assessed using the ROC curve shown in Figure 2. In the ROC curve analysis, the AUC was 0.619 (95% CI: 0.563–0.675), which is considered suitable for predictive purposes. The optimal cut-off threshold was 1.135. Based on this threshold, patients were dichotomized into the following two groups: groups with an OSI of ≥1.135 (defined as belonging to the high OSI category) and <1.135 (defined as belonging to the low OSI category).

### 3.3 Baseline characteristics

Among the 448 patients with DOR, 130 (29%) achieved clinical pregnancy. These patients were assigned to the clinical pregnancy and non-pregnancy groups. Supplementary Table S1 presents the baseline characteristics of each group. Statistically significant differences were observed between the groups in age, infertility type, COH protocol, number of oocytes, number of MII, OSI, fertilization protocol, number of zygotes observed with two pronuclei (2PN), embryo grade, and number of embryos (*p* < 0.05).

### 3.4 Univariate logistic regression analysis of predictors for clinical pregnancy

Supplementary Table S2 presents the univariate logistic regression analysis of clinical pregnancies in the modelling set. According to the analysis, clinical pregnancy in patients with expected DOR was associated with OSI, age, infertility type, BMI, AMH, COH protocol, number of oocytes, number of MII, fertilization protocol, number of 2PN, endometrium, embryo grade, and number of embryos (*p* < 0.1).

### 3.5 LASSO regression analysis of predictors for clinical pregnancy

The LASSO analysis was conducted on factors (including OSI, age, infertility type, BMI, AMH, COH protocol, number of oocytes, number of MII, fertilization protocol, number of 2PN, endometrium, embryo grade, and number of embryos), which were statistically significant (*p* < 0.1), as identified by univariate logistic regression. Supplementary Figure S1 presents the results. The optimal lambda value was determined based on the maximum lambda (lambda.1se), which falls within one standard error of the minimum cross-validation error (lambda.min). Supplementary Figure S1B shows the critical lambda values depicted as two vertical lines. Using this optimal lambda, the LASSO regression model selected the following seven predictors: OSI, age, COH, infertility type, fertilization protocol, number of oocytes, and number of embryos.

### 3.6 Multivariate logistic regression analysis and model visualization

**Figure 3:**
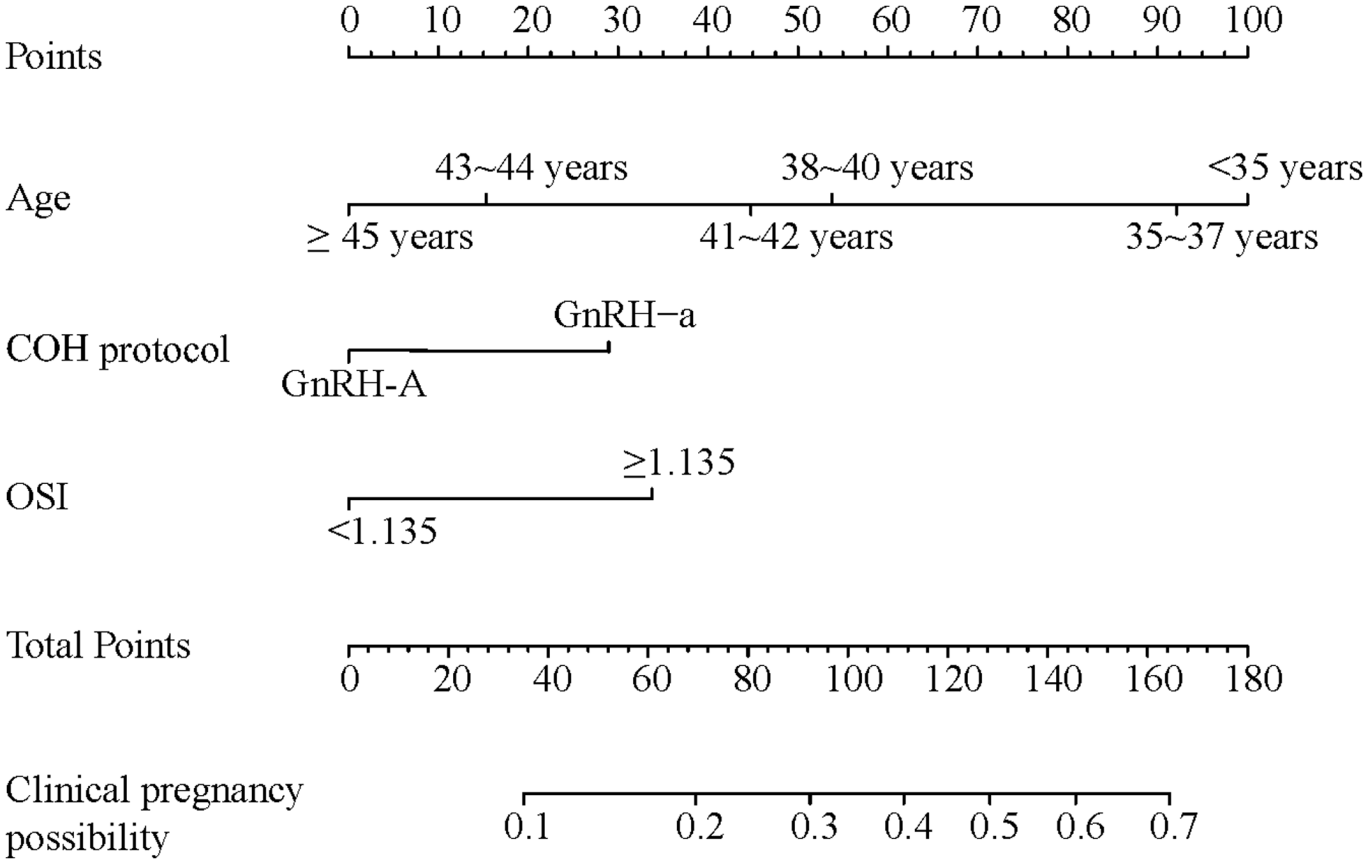
Nomograph plot based on the prediction model the value of each predictor was scored on a different point scale, after which the scores for each variable were added together. That sum is located on the total points axis, which corresponds to the clinical pregnancy possibility. For age categories, ≥45 years = 0, 43–44 years = 15, 41–42 years = 45, 38–40 years = 55, 35–37 years = 92.5, <35 years = 100. For COH protocol, GnRH-a protocol = 30, GnRH-A protocol = 0. For OSI, ≥1.135 = 35, <1.135 = 0. COH, controlled ovarian hyperstimulation; GnRH-a, gonadotropin-releasing hormone agonist; GnRH-A, gonadotropin-releasing hormone antagonist.

Multivariate logistic regression analysis was performed on the seven predictors screened using LASSO. Three predictors, OSI, age, and COH protocol, were finally included in the model according to the results shown in Table 1 (*p* < 0.05). The results of the logistic regression analyses were used to construct a nomogram to predict clinical pregnancy outcomes (Figure 3).

**Table 1:** Results of multivariate logistic regression analysis of predictors for clinical pregnancy.

Variable	B	SE	OR	CI	Z	*P*-value
Age 35–37 (years)	−0.226	0.334	0.8	0.41–1.54	−0.676	0.499
Age 38–40 (years)	−1.153	0.324	0.32	0.17–0.6	−3.553	<0.001^***^
Age 41–42 (years)	−1.358	0.362	0.26	0.13–0.52	−3.751	<0.001^***^
Age 43–44 (years)	−2.063	0.494	0.13	0.05–0.33	−4.171	<0.001^***^
Age ≥45 (years)	−2.405	0.572	0.09	0.03–0.28	−4.203	<0.001^***^
High OSI	0.595	0.272	1.81	1.06–3.09	2.193	0.028^*^
COH (Antagonist protocol)	−0.633	0.303	0.53	0.29–0.96	−2.09	0.037^*^
Fertilization protocol (ICSI)	−0.578	0.32	0.56	0.3–1.05	−1.809	0.07
Embryo grade (Low-quality)	−0.528	0.367	0.59	0.29–1.21	−1.439	0.15
No. of embryos	0.432	0.264	1.54	0.92–2.58	1.632	0.103

*P < 0.05; ***P < 0.001.

OSI, ovarian sensitivity index; COH, controlled ovarian hyperstimulation; ICSI, intracytoplasmic sperm injection; OR, odds ratio; B, coefficient; Z, z-value; SE, standard error; CI, confidence interval; no., number.

### 3.7 Model validation

#### 3.7.1 Discrimination ability of the nomogram model

**Figure 4:**
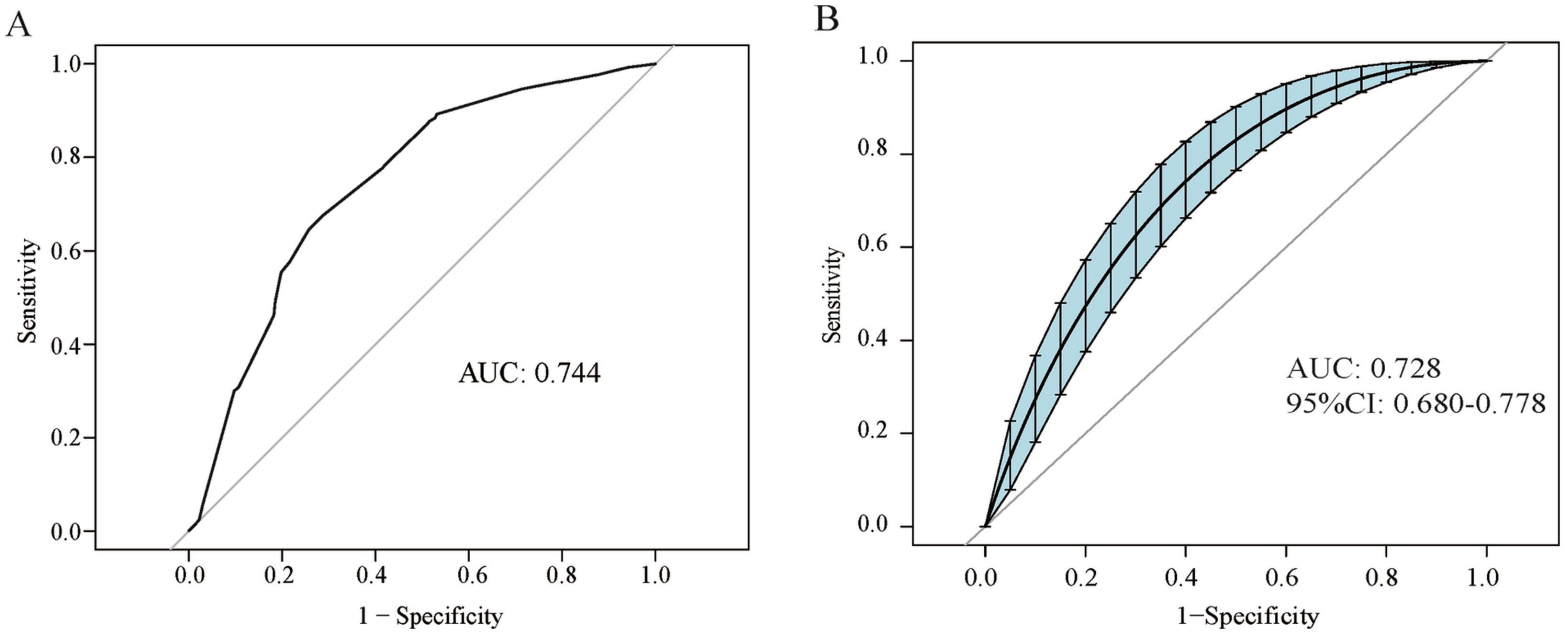
Discrimination ability of the nomogram model **(A)** ROC curve of the nomogram model. **(B)** ROC curve enhanced by 2,000 bootstrap replications. ROC, receiver operating characteristic; AUC, area under the curve; CI, confidence interval.

A ROC curve was constructed to demonstrate the predictive capability of the nomogram, with an AUC of 0.744 (Figure 4A). Internal validity, which assesses the reliability of the nomogram, was evaluated using 2,000 random bootstrap resamplings to mitigate overfitting bias. As shown in Figure 4B, the AUC of bootstrap resampling was 0.728 (95% CI: 0.680–0.778), suggesting that the nomogram exhibited satisfactory sensitivity.

#### 3.7.2 Prediction accuracy of the nomogram model

The bootstrap method, employing 2,000 repetitions, was used for internal validation. Supplementary Figure S2 displays the results of the H-L test and calibration curves. The H-L test yielded a chi-square statistic (X^2^) of 8.553 with a corresponding *p*-value of 0.480 (*p* > 0.05), indicating good agreement between the predictions and observations in the nomogram model. Additionally, the apparent and bias-corrected calibration curves of the nomogram model were closely aligned with the ideal curves, demonstrating robust agreement in the retrospective cohort.

#### 3.7.3 Clinical utility of the nomogram model

**Figure 5:**
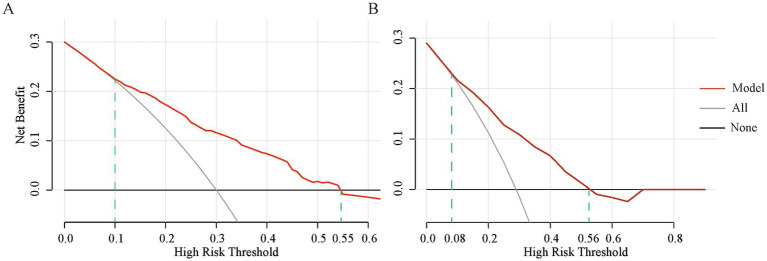
Clinical utility of the nomogram model **(A)** DCA curve of the baseline nomogram model. **(B)** DCA curve of the bootstrap-enhanced model. The red solid line represents the net benefit of using this model for decision-making at different threshold probabilities; the gray solid line indicates the net benefit when all patients are treated with IVF/ICSI based on a high assumed pregnancy probability; and the black-gray solid line represents the net benefit when no patients are treated with IVF/ICSI. DCA, Decision Curve Analysis; IVF/ICSI, *in vitro* fertilization/intracytoplasmic sperm injection.

Figure 5 presents the DCA of the nomogram model. This demonstrates that higher net clinical benefits are achievable with the baseline and bootstrap-enhanced models when their threshold probabilities range from 10 to 55% and 8 to 56%, respectively, than with scenarios where no patients or all patients undergo IVF/ICSI.

### 3.8 Birth outcomes

Among the 448 patients included in this study, 130 (29%) achieved clinical pregnancy. Of these, 96 (21.4%) resulted in live births, 30 (6.7%) ended in miscarriage, and 4 (0.89%) were ectopic pregnancies.

## 4 Discussion

In this study, we evaluated the predictive value of OSI, age, and the COH protocol for clinical pregnancy outcomes in patients with DOR undergoing IVF/ICSI. We also developed and internally validated a nomogram that integrated these variables, providing clinicians with a tool for making individualized treatment decisions and enhancing the pregnancy outcomes of IVF/ICSI procedures in the DOR cohort.

We selected clinical pregnancy rather than live birth as the endpoint outcome for the following reasons: First, the process from clinical pregnancy to live birth is influenced by many additional variables, including pregnancy complications, obstetric complications, fetal development, delivery mode, changes in maternal physical condition, and environmental factors. Although minimally associated with ovarian function and IVF/ICSI treatment efficacy in patients with DOR, these variables significantly affect the final live birth outcome. Second, clinical pregnancy (defined as the presence of an intrauterine gestational sac with fetal heartbeat at 28 days post-transfer) is a relatively stable and standardized evaluation indicator that can accurately reflect the early response of DOR patients to IVF/ICSI treatment. In our study, although the live birth rate was 21.4%, the miscarriage rate of 6.7% and ectopic pregnancy rate of 0.89% indicate that selecting clinical pregnancy as the primary outcome measure can better evaluate the early effects of IVF/ICSI treatment while avoiding interference from other factors in later stages, thus providing more targeted decision-making references for clinicians.

Studies have demonstrated that patients with a greater number of retrieved oocytes exhibit a higher cumulative pregnancy rate than those with fewer retrieved oocytes (20). Further studies have identified a significant correlation between the number of large follicles (diameter >17 mm), the number of retrieved oocytes, the number of high-quality embryos, and the clinical pregnancy rate (21). Consequently, assessing the ovarian response to stimulation in patients with DOR is crucial for predicting pregnancy outcomes of IVF/ICSI procedures. Common indicators used to assess ovarian responsiveness include AFC, AMH, and bFSH (22). AFC reflects the reserve of antral follicles in the ovaries; however, its accuracy is influenced by the skill of the operator and the menstrual cycle phase. The bFSH level is associated with the total amount of exogenous Gn administered, and a bFSH level >10 IU/L indicates poor ovarian response, although its predictive value is limited owing to variability (2, 23). AMH appears to reflect only the functional ovarian reserve, that is, the number of oocytes that could potentially mature in a given menstrual cycle, and does not directly indicate the size of the primordial follicle pool or the reproductive potential of the oocytes (24). Although AMH positively correlates with the number of embryos retrieved, it does not predict the clinical pregnancy rate in IVF/ICSI procedures (25). Furthermore, AMH levels can also be affected by factors such as BMI, ethnicity, smoking, environmental influences, diseases, and medications (26, 27).

This study selected the OSI as a predictive factor for pregnancy outcomes because of the potential confounding influences of various external factors and the inherent limitations of conventional markers. Prospective studies have shown that OSI has a strong negative correlation with AMH, and it may serve as an alternative predictor for ovarian response to exogenous Gn. Furthermore, the correlation between OSI and the number of oocytes retrieved was more pronounced than that between factors such as age, AFC, AMH, Gn dosage, and duration of ovarian hyperstimulation (28). Research has demonstrated that OSI more accurately predicts pregnancy outcomes than bFSH and AMH, particularly in patients with infertility aged >39 years, where OSI emerges as the optimal indicator for predicting cumulative pregnancy rates following IVF/ICSI treatments (29). In patients with DOR, OSI can predict embryo quality, clinical pregnancy rates, and live birth rates independent of AMH and bFSH levels (17). Consistent with previous studies, the results of the ROC curve analysis in our study suggested a relationship between OSI and clinical pregnancy outcomes in patients with DOR undergoing IVF/ICSI. In the univariate regression analysis, OSI variables—categorized using thresholds (1.135) derived from ROC curve analysis—revealed a significant difference in pregnancy outcomes between the high and low OSI groups (*p* < 0.001), indicating that OSI has a predictive value for pregnancy outcomes in the DOR population. Based on these results, the high OSI group exhibited a higher clinical pregnancy rate than the low OSI group. According to the OSI formula, a higher OSI indicates a lower total amount of Gn used and a higher number of oocytes retrieved, suggesting better ovarian responsiveness. This implies that within the DOR population, patients with better ovarian responsiveness are more likely to achieve clinical pregnancy than those without, which is consistent with other studies. It should be noted that because the OSI is derived from data on the number of oocytes retrieved and the total Gn dosage used in previous ART cycles, it cannot be applied to patients who have not yet undergone treatment. However, patients with DOR usually require multiple oocyte-retrieval cycles to achieve pregnancy. In cases where patients encounter limited oocyte numbers or fail IVF/ICSI, counselling about their reproductive potential and future treatment options is crucial. OSI represents the exogenous ovarian stimulation required for a specific patient to maximize oocyte yield and provides a precise and accurate measure. It incorporates not only the patient’s intrinsic characteristics but also their response to the COH protocol. This surpasses other ovarian responsiveness indicators, such as AFC, AMH, and bFSH, which merely qualitatively assess ovarian reserve function. Therefore, OSI holds the potential for predicting pregnancy outcomes, which facilitates better decision-making in subsequent IVF/ICSI cycles for patients with DOR. Notably, the interpretation of OSI should consider individual patient characteristics and clinic-specific protocols, as gonadotropin dosing strategies may vary between centers.

This study incorporates multiple variables into the analysis to construct a more comprehensive predictive model. Although multiple factors, including BMI, infertility type, and baseline hormone levels, may affect pregnancy outcomes, we carefully considered potential confounding factors through systematic variable selection. A rigorous three-step variable selection process was employed to develop a parsimonious and clinically meaningful prediction model. Initial univariate analysis with *p* < 0.1 identified 13 potential predictors, including OSI, age, infertility type, BMI, AMH, COH protocol, number of oocytes, number of MII oocytes, fertilization protocol, number of 2PN, endometrial thickness, embryo grade, and number of embryos. Subsequently, LASSO regression analyses helped address potential multicollinearity among these interrelated variables and selected seven key predictors: OSI, age, COH protocol, infertility type, fertilization protocol, number of oocytes, and number of embryos. The final multivariate model retained only three independent predictors with *p* < 0.05: OSI, age, and COH protocol. Several variables were excluded from the final model due to high correlation with included predictors or mediated effects through other variables in the model. The three final predictors represent distinct aspects: age reflects quantitative and qualitative ovarian reserve, OSI captures dynamic ovarian response, and COH protocol represents treatment strategy effects. Clinical studies indicate that younger patients with DOR do not experience a decline in fertility during IVF/ICSI treatments but have a reduced number of oocytes retrieved (30, 31). Another study shows that although younger patients with DOR have higher rates of mature oocytes, normal fertilization, high-quality embryos, and clinical pregnancy than older patients with DOR, their outcomes are still inferior to those of younger women with normal ovarian reserve (32). For older patients with DOR, age-related decline in the quantity and quality of oocytes significantly impacts the adverse outcomes of IVF/ICSI (33). Studies have demonstrated that younger patients with DOR using the GnRH-a long protocol have lower cycle cancellation rates and higher implantation and live birth rates than those using the GnRH-A protocols (34). However, for older patients, the differences in pregnancy outcomes between these protocols are minimal. Although the long protocol provides advantages in terms of pregnancy rates, it may also lead to excessive pituitary suppression and an increased risk of adverse reactions (35). The results of these studies support our findings.

The nomogram demonstrates the relative contribution and clinical significance of each of the following predictors. (a) Age shows the strongest weight in the model, with points ranging from 0 to 100. This reflects the biological impact of aging on oocyte quantity and quality in patients with DOR. Specifically, patients aged <35 years receive the highest points (100), indicating optimal pregnancy potential. Points decrease progressively with increasing age, particularly after 38 years. Patients aged ≥45 years receive 0 points, reflecting significantly reduced pregnancy chances. This aligns with previous findings showing that age-related decline in IVF/ICSI outcomes is more pronounced in patients with DOR than in those without. (b) OSI contributes moderately (0–35 points): high OSI (≥1.135) receives 35 points, indicating better ovarian responsiveness, and low OSI (<1.135) receives 0 points. This quantifies the impact of ovarian response on pregnancy outcomes, with higher OSI reflecting more efficient stimulation and better oocyte yield per gonadotropin dose. (c) COH protocol has the smallest but still significant weight (0–30 points): GnRH-a protocol receives 30 points, and GnRH-A protocol receives 0 points. This difference reflects the documented advantages of GnRH-a protocols in patients with DOR, particularly in younger age groups. According to the final nomogram, older patients with DOR with low OSI and those undergoing the GnRH-A protocols had decreased clinical pregnancy rates. To use this nomogram in clinical practice, clinicians can calculate points for each predictor (age, OSI, and COH protocol) and sum up the total points to determine the corresponding predicted clinical pregnancy probability. For example, a DOR patient aged 34 years (100 points) with high OSI < 1.135 (0 points) using GnRH-a protocol (30 points) would have a total of 130 points, corresponding to approximately 50% probability of clinical pregnancy.

We conducted a model evaluation to further assess the stability and reliability of the nomogram model. Given the small sample size and low clinical pregnancy rates in patients with DOR, to minimize bias, we applied the bootstrap internal validation method to evaluate the model in the following three dimensions: discrimination, calibration, and clinical utility. Discrimination was assessed through ROC curve analysis, with both the nomogram model and the internally validated ROC curves achieving an AUC of >0.7, indicating that the model effectively distinguished between different pregnancy outcomes. Calibration was evaluated using calibration curves, and the H–L test revealed no statistical difference between the actual clinical pregnancy outcomes and those predicted by the nomogram model. Furthermore, the calibration curves of the nomogram model, ideal calibration curve, and bias-corrected curve overlapped closely, demonstrating the accuracy of the predictions of the model. Clinical utility was assessed using DCA curves, with both the nomogram model and the post-internal validation DCA curves, showing that using this model to predict clinical pregnancy may yield more benefits, particularly when considering threshold probabilities ranging from 10 to 55% and 8 to 56%. This indicates that the nomogram model has a wide range of applications.

Although our study shows promising results, it still has some limitations, including a small sample size, single-center data, and the absence of external validation. We acknowledge that multicenter validation would further strengthen the generalizability of the model. Currently, we are establishing collaborations with other reproductive centers to validate our nomogram in different populations. Future studies should focus on multi-center validation across different geographic regions, validation in diverse ethnic populations, prospective validation to confirm the predictive accuracy of the model, and assessment of the performance of the model in different clinical settings.

Another limitation is that we chose clinical pregnancy rather than live birth as the endpoint. Although clinical pregnancy can better reflect the early response to IVF/ICSI treatment while avoiding interference from later factors, it may not fully represent the ultimate reproductive outcome that patients desire the most. The progression from clinical pregnancy to live birth involves multiple additional variables that could affect the final outcome. Future studies may benefit from incorporating both clinical pregnancy and live birth outcomes to provide more comprehensive prognostic information.

In conclusion, a user-friendly and effective nomogram was developed in this study to predict the clinical pregnancy outcomes during IVF/ICSI procedures in patients with DOR. This nomogram serves as a valuable tool for the personalized prediction and improvement of pregnancy outcomes in patients with DOR.

## Data Availability

The data analyzed in this study are subject to the following licenses/restrictions: the datasets used and/or analyzed during the current study are available from the corresponding author on reasonable request. Requests to access these datasets should be directed to liufengxia34@163.com.

## Glossary

AMH: Anti-Müllerian hormone
AFC: Antral follicle count
AUC: Area under the ROC curve
ART: Assisted reproductive technologies
bFSH: Baseline follicle-stimulating hormone
BMI: Body mass index
CI: Confidence interval
COH: Controlled ovarian hyperstimulation
DCA: Decision curve analysis
DOR: Diminished ovarian reserve
Gn: Gonadotropin
GnRH-a: Gonadotropin-releasing hormone agonist
GnRH-A: Gonadotropin-releasing hormone antagonist
H-L: Hosmer–Lemeshow
HCG: Human Chorionic Gonadotropin
IVF/ICSI: *In vitro* fertilization/intracytoplasmic sperm injection
LASSO: Least absolute shrinkage and selection operator
LH: Luteinizing hormone
MII: Metaphase II
OSI: Ovarian sensitivity index
ROC: Receiver operating characteristic

## References

[1] Pastore LM Christianson MS Stelling J Kearns WG Segars JH. Reproductive ovarian testing and the alphabet soup of diagnoses: DOR, POI, POF, POR, and FOR. J Assist Reprod Genet. (2018) 35:17–23. doi: 10.1007/s10815-017-1058-4, PMID: 28971280 PMC5758472

[2] Practice Committee of the American Society for Reproductive Medicine. Testing and interpreting measures of ovarian reserve: a committee opinion. Fertil Steril. (2020) 114:1151–1157. doi: 10.1016/j.fertnstert.2020.09.134 33280722

[3] Erdoğan K Şanlıer N Utlu E Güvey H Kahyaoğlu İ Neşelioğlu S. Serum and follicular fluid thiol/disulfide homeostasis in diminished ovarian reserve patients undergoing in vitro fertilization/intracytoplasmic sperm injection treatment. Cureus. (2023) 15:e35476. doi: 10.7759/cureus.35476, PMID: 36855584 PMC9968409

[4] Jin H Yan E Chen D Zhao M Peng W Guo Y. Diminished ovarian reserve may not be associated with a poorer fresh cycle outcome in women <38 years. J Ovarian Res. (2023) 16:77. doi: 10.1186/s13048-023-01158-6, PMID: 37061732 PMC10105451

[5] Buratini J Dal Canto M De Ponti E Brambillasca F Brigante C Gippone S. Maternal age affects the relationship of basal FSH and anti-Müllerian hormone concentrations with post-ICSI/IVF live birth. Reprod Biomed Online. (2021) 42:748–56. doi: 10.1016/j.rbmo.2020.12.005, PMID: 33653653

[6] Chen H Li J Cai S Zeng S Yin C Kuang W. Impact of body mass index (BMI) on the success rate of fresh embryo transfer in women undergoing first in vitro fertilization/intracytoplasmic sperm injection (IVF/ICSI) treatment. Int J Obes. (2022) 46:202–10. doi: 10.1038/s41366-021-00978-0, PMID: 34628467

[7] Barreto CN Castro GZ Pereira RG Pereira FAN Reis FM Junior WM. Predicting *in vitro* fertilization success in the Brazilian public health system: a machine learning approach. Med Biol Eng Comput. (2022) 60:1851–61. doi: 10.1007/s11517-022-02569-1 35508786

[8] Ratna MB Bhattacharya S McLernon DJ. External validation of models for predicting cumulative live birth over multiple complete cycles of IVF treatment. Hum Reprod. (2023) 38:1998–2010. doi: 10.1093/humrep/dead165, PMID: 37632223 PMC10546080

[9] Chen Y Niu A Feng X Zhang Y Li F. Prediction of pregnancy outcome in fresh in vitro fertilization/intracytoplasmic sperm injection treatment in patients with poor ovarian reserve. Aging (Albany NY). (2021) 13:18331–9. doi: 10.18632/aging.203282, PMID: 34273144 PMC8351725

[10] Chen Z Li W Ma S Li Y Lv L Huang K. Evaluative effectiveness of follicular output rate, ovarian sensitivity index, and ovarian response prediction index for the ovarian reserve and response of low-prognosis patients according to the POSEIDON criteria: a retrospective study. Zygote. (2023) 31:557–69. doi: 10.1017/S0967199423000382, PMID: 37737063

[11] Feng K Zhang Z Wu L Zhu L Li X Li D. Predictive factors for the formation of viable embryos in subfertile patients with diminished ovarian reserve: a clinical prediction study. Reprod Sci. (2024) 31:1747–56. doi: 10.1007/s43032-024-01469-z, PMID: 38409494 PMC11111567

[12] Zhu S Jiang W Liao X Sun Y Chen X Zheng B. Effect of diminished ovarian reserve on the outcome of fresh embryo transfer in IVF/ICSI cycles among young women: a retrospective cohort study. BMC Womens Health. (2024) 24:230. doi: 10.1186/s12905-024-03039-6, PMID: 38594688 PMC11003098

[13] He Y Liu L Yao F Sun C Meng M Lan Y. Assisted reproductive technology and interactions between serum basal FSH/LH and ovarian sensitivity index. Front Endocrinol. (2023) 14:1086924. doi: 10.3389/fendo.2023.1086924, PMID: 37206442 PMC10190590

[14] Revelli A Gennarelli G Biasoni V Chiadò A Carosso A Evangelista F. The ovarian sensitivity index (OSI) significantly correlates with ovarian reserve biomarkers, is more predictive of clinical pregnancy than the total number of oocytes, and is consistent in consecutive IVF cycles. J Clin Med. (2020) 9:1914. doi: 10.3390/jcm9061914, PMID: 32570935 PMC7355532

[15] Zhuang GL. Advanced assisted reproduction techniques. Beijing: People’s Medical Publishing House (2005).

[16] Gardner DK Schoolcraft WB. Culture and transfer of human blastocysts. Curr Opin Obstet Gynecol. (1999) 11:307–11. doi: 10.1097/00001703-199906000-00013, PMID: 10369209

[17] Weghofer A Barad DH Darmon SK Kushnir VA Albertini DF Gleicher N. The ovarian sensitivity index is predictive of live birth chances after IVF in infertile patients. Hum Reprod Open. (2020) 2020:hoaa049. doi: 10.1093/hropen/hoaa049, PMID: 33381657 PMC7753003

[18] Hu L Bu Z Huang G Sun H Deng C Sun Y. Assisted Reproductive Technology in China: Results Generated From Data Reporting System by CSRM From 2013 to 2016. Front Endocrinol. (2020) 11:458. doi: 10.3389/fendo.2020.00458 PMC7527788 33042000

[19] WHO Expert Consultation. Appropriate body-mass index for Asian populations and its implications for policy and intervention strategies. Lancet. (2004) 363:157–63. doi: 10.1016/S0140-6736(03)15268-3, PMID: 14726171

[20] Alvaro Mercadal B Rodríguez I Arroyo G Martínez F Barri PN Coroleu B. Characterization of a suboptimal IVF population and clinical outcome after two IVF cycles. Gynecol Endocrinol. (2018) 34:125–8. doi: 10.1080/09513590.2017.1369515, PMID: 28868939

[21] Özelçi R Aldemir O Dilbaz S Özkaya E Kahyaoğlu İ Dilbaz B. The impact of different etiologies of diminished ovarian reserve on pregnancy outcome in IVF-ET cycles. Turk J Med Sci. (2019) 49:1138–44. doi: 10.3906/sag-1811-175, PMID: 31293144 PMC7018253

[22] Lensen SF Wilkinson J Leijdekkers JA La Marca A Mol BWJ Marjoribanks J. Individualised gonadotropin dose selection using markers of ovarian reserve for women undergoing in vitro fertilisation plus intracytoplasmic sperm injection (IVF/ICSI). Cochrane Database Syst Rev. (2018) 2018:CD012693. doi: 10.1002/14651858.CD012693.pub2, PMID: 29388198 PMC6491064

[23] La Marca A Sunkara SK. Individualization of controlled ovarian stimulation in IVF using ovarian reserve markers: from theory to practice. Hum Reprod Update. (2014) 20:124–40. doi: 10.1093/humupd/dmt037 24077980

[24] Moolhuijsen LME Visser JA. Anti-Müllerian hormone and ovarian reserve: update on assessing ovarian function. J Clin Endocrinol Metab. (2020) 105:3361–73. doi: 10.1210/clinem/dgaa513, PMID: 32770239 PMC7486884

[25] Li L Sun B Wang F Zhang Y Sun Y. Which factors are associated with reproductive outcomes of DOR patients in ART cycles: an eight-year retrospective study. Front Endocrinol. (2022) 13:796199. doi: 10.3389/fendo.2022.796199, PMID: 35813637 PMC9259947

[26] Ding X Kou X Zhang Y Zhang X Cheng G Jia T. Leptin siRNA promotes ovarian granulosa cell apoptosis and affects steroidogenesis by increasing NPY2 receptor expression. Gene. (2017) 633:28–34. doi: 10.1016/j.gene.2017.08.028, PMID: 28864114

[27] Moslehi N Shab-Bidar S Ramezani Tehrani F Mirmiran P Azizi F. Is ovarian reserve associated with body mass index and obesity in reproductive aged women? A meta-analysis. Menopause. (2018) 25:1046–55. doi: 10.1097/GME.0000000000001116, PMID: 29738413

[28] Li HW Lee VC Ho PC Ng EH. Ovarian sensitivity index is a better measure of ovarian responsiveness to gonadotrophin stimulation than the number of oocytes during in-vitro fertilization treatment. J Assist Reprod Genet. (2014) 31:199–203. doi: 10.1007/s10815-013-0144-5 24317853 PMC3933607

[29] Cesarano S Pirtea P Benammar A De Ziegler D Poulain M Revelli A. Are there ovarian responsive indexes that predict cumulative live birth rates in women over 39 years? J Clin Med. (2022) 11:2099. doi: 10.3390/jcm11082099, PMID: 35456190 PMC9031602

[30] Morin SJ Patounakis G Juneau CR Neal SA Scott RT Seli E. Diminished ovarian reserve and poor response to stimulation in patients <38 years old: a quantitative but not qualitative reduction in performance. Hum Reprod. (2018) 33:1489–98. doi: 10.1093/humrep/dey238, PMID: 30010882

[31] Yao LN Lin WQ Jiang N Li C Cao HF Li H. Comparative study of assisted reproductive outcomes between young patients with occult premature ovarian insufficiency and advanced-age patients. J Int Med Res. (2020) 48:300060520934656. doi: 10.1177/0300060520934656, PMID: 32586157 PMC7323297

[32] Chang Y Li J Li X Liu H Liang X. Egg quality and pregnancy outcome in young infertile women with diminished ovarian reserve. Med Sci Monit. (2018) 24:7279–84. doi: 10.12659/MSM.910410, PMID: 30310048 PMC6195787

[33] Devesa M Tur R Rodríguez I Coroleu B Martínez F Polyzos NP. Cumulative live birth rates and number of oocytes retrieved in women of advanced age. A single Centre analysis including 4500 women ≥38 years old. Hum Reprod. (2018) 33:2010–7. doi: 10.1093/humrep/dey295, PMID: 30272168

[34] Huang MC Tzeng SL Lee CI Chen HH Huang CC Lee TH. GnRH agonist long protocol versus GnRH antagonist protocol for various aged patients with diminished ovarian reserve: a retrospective study. PLoS One. (2018) 13:e0207081. doi: 10.1371/journal.pone.0207081, PMID: 30403766 PMC6221355

[35] Papamentzelopoulou M Stavros S Mavrogianni D Kalantzis C Loutradis D Drakakis P. Meta-analysis of GnRH-antagonists versus GnRH-agonists in poor responder protocols. Arch Gynecol Obstet. (2021) 304:547–57. doi: 10.1007/s00404-020-05954-z, PMID: 33423109

