# Supplementary material for: Ovarian sensitivity index-based nomogram for predicting clinical pregnancy outcomes in patients with diminished ovarian reserve undergoing in vitro fertilization or intracytoplasmic sperm injection
Source: Front Med (Lausanne). 2025 Jun 27;12:1618552. doi: 10.3389/fmed.2025.1618552 (PMC12245897; doi:10.3389/fmed.2025.1618552)
Supplement: Supplementary file 1 [file Table_1.DOCX]

**Table S1. Baseline characteristics of patients with diminished ovarian reserve undergoing IVF/ICSI**

| Variable | All patients  N (%) | Clinical pregnancy  N (%) | Non-pregnancy  N (%) | P-value |
| --- | --- | --- | --- | --- |
|  | 448 | 130 (29) | 318 (71) |  |
| Age (years) |  |  |  | <0.001^***^ |
| <35 | 88 (19.6) | 44 (33.8) | 44 (13.8) |  |
| 35–37 | 71 (15.8) | 33 (25.4) | 38 (11.9) |  |
| 38–40 | 105 (23.4) | 26 (20.0) | 79 (24.8) |  |
| 41–42 | 80 (17.9) | 17 (13.1) | 63 (19.8) |  |
| 43–44 | 54 (12.1) | 6 (4.62) | 48 (15.1) |  |
| ≥45 | 50 (11.2) | 4 (3.08) | 46 (14.5) |  |
| BMI (kg/m^2^) |  |  |  | 0.098 |
| <18.5 | 217 (48.4) | 71 (54.6) | 146 (45.9) |  |
| 18.5–23 | 15 (3.3.5) | 6 (4.62) | 9 (2.83) |  |
| ≥23 | 216 (48.2) | 53 (40.8) | 163 (51.3) |  |
| AMH level (ng/mL) | 0.53 ± 0.25 | 0.56 ± 0.26 | 0.51 ± 0.25 | 0.063 |
| bFSH level (U/L) | 11.7 ± 5.99 | 11.2 ± 6.23 | 11.8 ± 5.89 | 0.343 |
| Infertility type |  |  |  | 0.03^*^ |
| Primary | 99 (22.1) | 39 (30.0) | 60 (18.9) |  |
| Secondary | 327 (73.0) | 84 (64.6) | 243 (76.4) |  |
| Other | 22 (4.91) | 7 (5.38) | 15 (4.72) |  |
| COH protocol |  |  |  | 0.004^**^ |
| GnRH-a protocol | 62 (13.8) | 28 (21.5) | 34 (10.7) |  |
| GnRH-A protocol | 386 (86.2) | 102 (78.5) | 284 (89.3) |  |
| Total Gn dose | 2878 ± 1042 | 2869 ± 955 | 2881 ± 1077 | 0.906 |
| No. of oocytes | 4.35 ± 2.60 | 5.08 ± 2.62 | 4.05 ± 2.53 | <0.001^***^ |
| No. of MII | 3.75 ± 2.34 | 4.35 ± 2.37 | 3.51 ± 2.29 | 0.001^***^ |
| OSI |  |  |  | <0.001^***^ |
| High | 288 (64.3) | 101 (77.7) | 187 (58.8) |  |
| Low | 160 (35.7) | 29 (22.3) | 131 (41.2) |  |
| Fertilization protocol |  |  |  | 0.03^*^ |
| IVF | 359 (80.1) | 113 (86.9) | 246 (77.4) |  |
| ICSI | 89 (19.9) | 17 (13.3) | 72 (22.6) |  |
| No. of PN | 2.77 ± 1.75 | 3.17 ± 1.90 | 2.6 ± 1.66 | 0.003^**^ |
| Endometrium (mm) | 9.39 ± 2.10 | 9.76 ± 1.99 | 9.28 ± 2.14 | 0.065 |
| Embryo stage |  |  |  | 0.221 |
| D3 | 394 (87.9) | 110 (84.6) | 284 (89.3) |  |
| D5/D6 | 54 (12.1) | 20 (15.4) | 34 (10.7) |  |
| Embryo grade |  |  |  | 0.036^*^ |
| Good-quality | 380 (84.8) | 118 (90.8) | 262 (82.4) |  |
| Low-quality | 68 (15.2) | 12 (9.23) | 56 (17.6) |  |
| No. of embryos | 1.62 ± 0.49 | 1.72 ± 0.45 | 1.58 ± 0.50 | 0.004^**^ |

Continuous variables with a normal distribution are presented as mean ± standard deviation and were compared using the t-test. Categorical variables are expressed as proportion and were compared using the chi-square test. *P < 0.05, **P < 0.01, ***P < 0.001.

IVF, *in vitro* fertilization; ICSI, intracytoplasmic sperm injection; BMI, body mass index; COH, controlled ovarian hyperstimulation; GnRH-a, gonadotropin-releasing hormone agonist; GnRH-A, gonadotropin-releasing hormone antagonist; Gn, gonadotropin; MII, metaphase II; PN, pronuclei; D, day; AMH, anti-Müllerian hormone; bFSH, basal follicle-stimulating hormone; OSI, ovarian sensitivity index; No., number.

**Table S2. Results of univariate logistic regression analysis of the modeling set**

| Variable | B | SE | OR | CI | Z | P-value |
| --- | --- | --- | --- | --- | --- | --- |
| Age 35–37 (years) | −0.141 | 0.319 | 0.87 | 0.46–1.62 | −0.442 | 0.659 |
| Age 38–40 (years) | −1.111 | 0.311 | 0.33 | 0.18–0.61 | −3.576 | <0.001^***^ |
| Age 41–42 (years) | −1.31 | 0.347 | 0.27 | 0.14–0.53 | −3.779 | <0.001^***^ |
| Age 43–44 (years) | −2.079 | 0.483 | 0.12 | 0.05–0.32 | −4.308 | <0.001^***^ |
| Age ≥45 (years) | −2.442 | 0.563 | 0.09 | 0.03–0.26 | −4.337 | <0.001^***^ |
| BMI <18.5 (kg/m^2^) | 0.315 | 0.547 | 1.37 | 0.47–4.01 | 0.577 | 0.564 |
| BMI ≥23 (kg/m^2^) | −0.403 | 0.214 | 0.67 | 0.44–1.02 | −1.878 | 0.06' |
| AMH level (ng/mL) | 0.764 | 0.409 | 2.15 | 0.96–4.79 | 1.868 | 0.062' |
| bFSH level (U/L) | −0.017 | 0.018 | 0.98 | 0.95–1.02 | −0.972 | 0.331 |
| Infertility type (Secondary) | −0.631 | 0.242 | 0.53 | 0.33–0.85 | −2.615 | 0.009^**^ |
| Infertility type (Other) | −0.331 | 0.502 | 0.72 | 0.27–1.92 | -0.66 | 0.509 |
| COH (Antagonist protocol) | −0.830 | 0.280 | 0.44 | 0.25–0.75 | −2.963 | 0.003^**^ |
| Total Gn dose | 0.000 | 0.000 | 1.00 | 1.00–1.00 | −0.113 | 0.91 |
| No. of oocytes | 0.149 | 0.040 | 1.16 | 1.07–1.26 | 3.752 | <0.001^***^ |
| No. of MII | 0.147 | 0.043 | 1.16 | 1.07–1.26 | 3.386 | 0.001^***^ |
| High OSI | 0.892 | 0.240 | 2.44 | 1.52–3.91 | 3.724 | <0.001^***^ |
| Fertilization protocol (ICSI) | −0.666 | 0.293 | 0.51 | 0.29–0.91 | −2.274 | 0.023^*^ |
| No. of PN | 0.178 | 0.058 | 1.19 | 1.07–1.34 | 3.067 | 0.002^**^ |
| Endometrium (mm) | 0.090 | 0.05 | 1.09 | 0.99–1.21 | 1.793 | 0.073' |
| Embryo stage (D5/D6) | 0.418 | 0.303 | 1.52 | 0.84–2.75 | 1.377 | 0.168 |
| Embryo grade (Low-quality) | −0.743 | 0.337 | 0.48 | 0.25–0.92 | −2.205 | 0.027^*^ |
| No. of embryos | 0.617 | 0.225 | 1.85 | 1.19–2.88 | 2.744 | 0.006^**^ |

'P < 0.1, *P < 0.05, **P < 0.01, ***P < 0.001

BMI, body mass index; COH, controlled ovarian hyperstimulation; AMH, anti-Müllerian hormone; bFSH, basal follicle-stimulating hormone; Gn, gonadotropin; MII, metaphase II; PN, pronuclei; D, day; OSI, ovarian sensitivity index; ICSI, intracytoplasmic sperm injection; OR, odds ratio; B, coefficient; Z, z-value; SE, standard error; CI, confidence interval; No., number.
