# Supplementary figures and images for: Ovarian sensitivity index-based nomogram for predicting clinical pregnancy outcomes in patients with diminished ovarian reserve undergoing in vitro fertilization or intracytoplasmic sperm injection
Source: Front Med (Lausanne). 2025 Jun 27;12:1618552. doi: 10.3389/fmed.2025.1618552 (PMC12245897; doi:10.3389/fmed.2025.1618552)

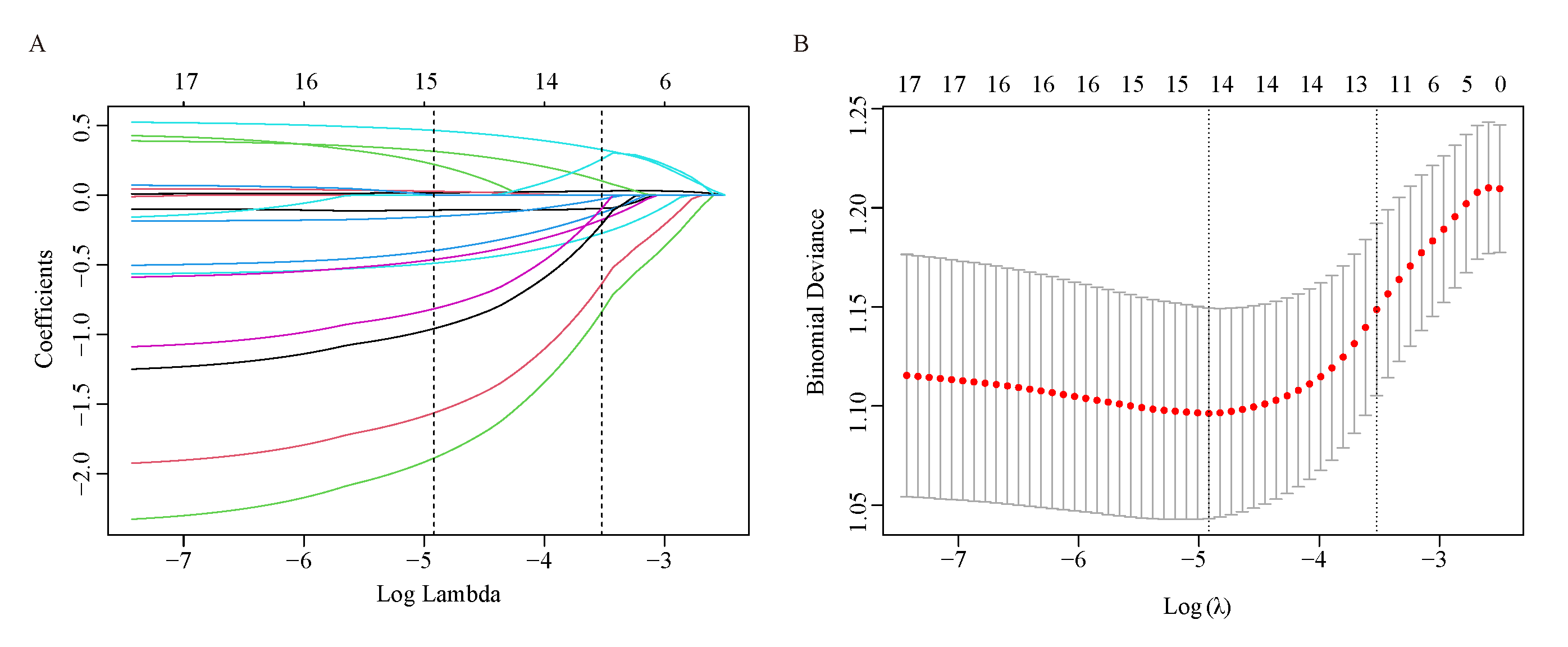

Supplement: Supplementary file 2 [file Image_1.TIFF]

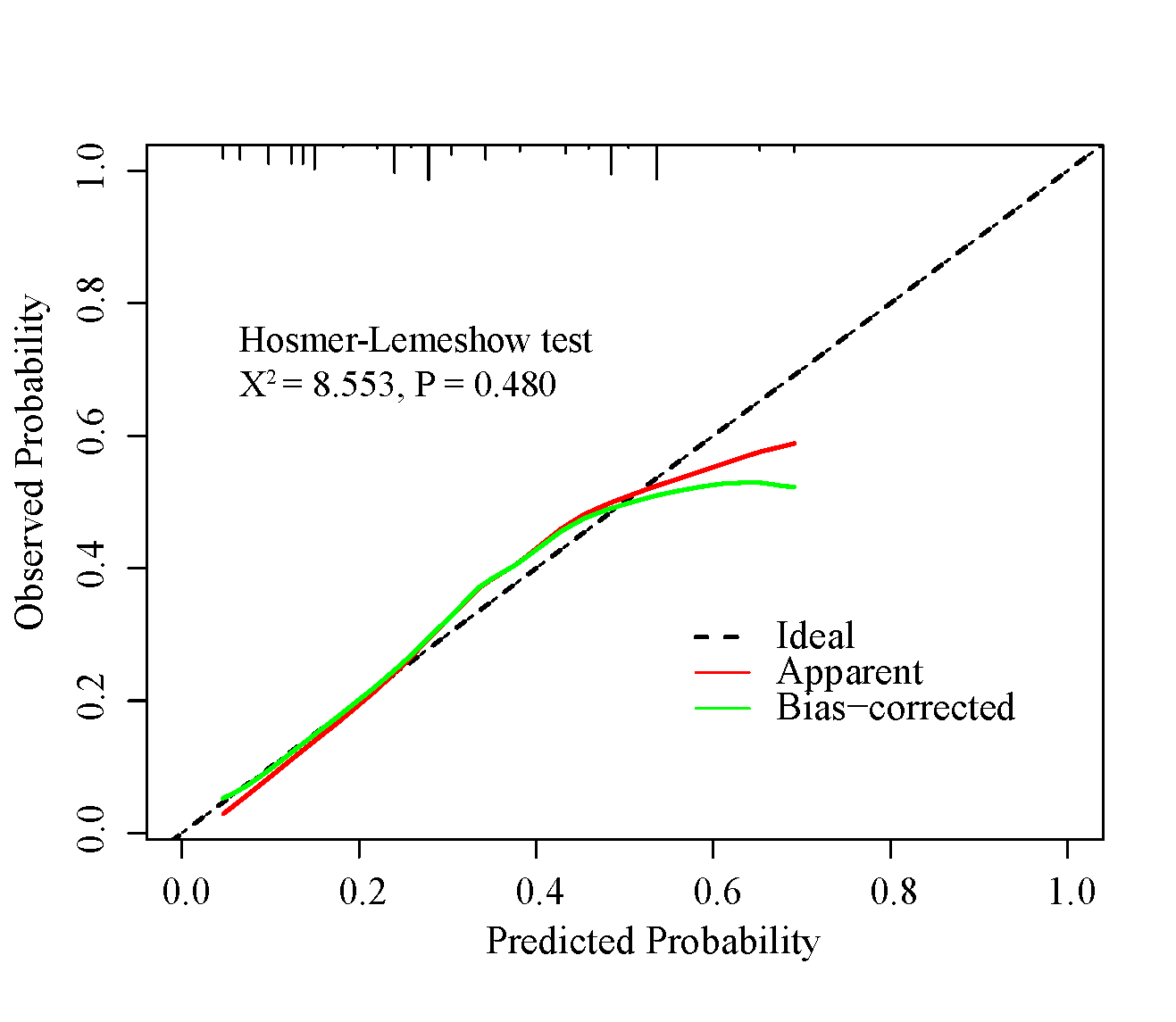

Supplement: Supplementary file 3 [file Image_2.TIFF]
